# Cytology in the diagnosis of cervical cancer in symptomatic young women: a retrospective review

**DOI:** 10.3399/bjgp16X687937

**Published:** 2016-10-25

**Authors:** Anita WW Lim, Rebecca Landy, Alejandra Castanon, Antony Hollingworth, Willie Hamilton, Nick Dudding, Peter Sasieni

**Affiliations:** Centre for Cancer Prevention, Wolfson Institute of Preventive Medicine, Queen Mary University, London.; Centre for Cancer Prevention, Wolfson Institute of Preventive Medicine, Queen Mary University, London.; Centre for Cancer Prevention, Wolfson Institute of Preventive Medicine, Queen Mary University, London.; Department of Gynaecology, Whipps Cross University Hospital, Barts Health NHS Trust, London.; Department of Primary Care, St Luke’s Campus, University of Exeter, Exeter.; East Pennine Cytology Training Centre, Raynham House, Leeds.; Centre for Cancer Prevention, Wolfson Institute of Preventive Medicine, Queen Mary University, London.

**Keywords:** cervical cancer, cervical cytology, early diagnosis, gynaecological symptoms, retrospective study, young women

## Abstract

**Background:**

Cervical cancer in young women presents a diagnostic challenge because gynaecological symptoms are common but underlying disease is rare.

**Aim:**

To explore the potential for using cytology as a diagnostic aid for cervical cancer in young women.

**Design and setting:**

Retrospective review of primary care records and cytology data from the national cervical screening database and national audit of cervical cancers.

**Method:**

Four datasets of women aged 20–29 years in England were examined: primary care records and national screening data from an in-depth study of cervical cancers; cytology from the national audit of cervical cancers; whole-population cytology from the national screening database; and general-population primary care records from the Clinical Practice Research Datalink. The authors explored the sensitivity and positive predictive value (PPV) of symptomatic cytology (earliest <12 months before diagnosis) to cervical cancer.

**Results:**

The estimated prevalence of cervical cancer among symptomatic women was between 0.4% and 0.9%. The sensitivity of moderate dyskaryosis (high-grade squamous intraepithelial lesion [HSIL]) or worse in women aged 20–29 years was 90.9% to 96.2% across datasets, regardless of symptom status. The PPV was estimated to be between 10.0% and 30.0%. For women aged 20–24 years, the PPV of ‘?invasive squamous carcinoma’ was 25.4%, and 2.0% for severe or worse cytology.

**Conclusion:**

Cytology has value beyond screening, and could be used as a diagnostic aid for earlier detection of cervical cancer in young women with gynaecological symptoms by ruling in urgent referral.

## INTRODUCTION

Cervical cancer in women aged <30 years is rarely fatal, but prompt diagnosis of young women with symptomatic cervical cancer remains an unmet need, and reports of delayed diagnosis continue to cause concern.^1^^–^3 In England, the age of first cervical screening changed from 20 years to 25 years in 2003. Because routine cervical screening is not recommended for women aged <25 years,^4^ and screening coverage among women aged 25–29 years is low (63% in England in 2013),^5^ diagnosis of cervical cancer in young women often relies on symptomatic presentation.

Symptoms of cervical cancer are also common in young women who have genital infections or are using hormonal contraceptives.^6^^,^^7^ An audit of primary care records in England found that over a period of 1 year, up to 1.6% of women aged 15–29 years presented with intermenstrual bleeding, 0.5% with postcoital bleeding, and 1.3% with vaginal discharge.^8^ Primary care clinicians are faced with the diagnostic challenge of identifying a rare disease (UK incidence rates of 3.3 and 19.8 per 100 000 women-years at ages 20–24 years and 25–29 years, respectively [2011 to 2013])^9^ on the basis of common symptoms.

The issue of how best to manage common gynaecological symptoms in women too young to be invited for routine screening is controversial, and has been debated in the UK Parliament.^10^ The main recommendation in UK guidelines is to visualise the cervix.^11^^–^13 However, this is based on expert opinion and lacks empirical evidence. The authors recently demonstrated that cervical examination missed the majority of cervical cancers, including stage 1B or worse.^14^ Furthermore, microinvasive cancers are not visible to the naked eye and are potentially symptomatic,^15^ and it is at this early stage that physicians would like to be able to diagnose cancers. Collectively these issues highlight the need for additional triage tools for primary care physicians managing young women with gynaecological symptoms.

Cervical cytology is well established as a screening test to detect pre-invasive cervical lesions (high-grade cervical intraepithelial neoplasia), but has rarely been explored as a test to detect cervical cancer. In fact, cytology has been discouraged in symptomatic young women because of concern over delays while awaiting results, and false reassurance if it is negative.^16^^,^^17^ This study investigates whether cytology could be used as a diagnostic aid to facilitate earlier diagnosis of cervical cancer in women aged 20–29 years with symptoms.

## METHOD

The study aimed to assess the diagnostic validity of cytology in the identification of invasive cervical cancer by estimating its sensitivity, specificity, and positive predictive value (PPV) in young women with relevant gynaecological symptoms (Box 1). No single source of data reports both the frequency of symptoms in the general population and the cytology results among the symptomatic population, therefore multiple sources (four) were necessary.

**How this fits in**

Cervical cancer in young women represents a diagnostic challenge because gynaecological symptoms are common but disease is rare. Cytology has a well-established role in cervical cancer prevention but not in diagnosis. This study shows that cytology at a threshold of moderate dyskaryosis or worse is an excellent diagnostic aid for detecting cervical cancer in young women with symptoms.

### Data sources

The four datasets included:

- primary care records and national cervical screening data from an in-depth study of cervical cancers;
- cytology from the national audit of cervical cancers;
- whole-population cytology from the national screening database; and
- general-population primary care records from the Clinical Practice Research Datalink.

Primary care records (symptoms) and national cervical screening data (cytology) from an in-depth study of women aged 18–29 years with cervical cancer, diagnosed between November 2010 and March 2012,^15^ provided estimates of sensitivity, and indirectly of PPV. The original study focused on interview data that have been reported elsewhere.^15^ Primary care records were available for 107 (84.0%) of 128 women recruited, and cervical screening data for 112 (88.0%). The age range of these 107 women was 22–29 years. The entire primary care record was used to identify gynaecological symptomatic presentations in the year before diagnosis (Box 1). In the in-depth study, the authors defined symptomatic cytology tests as those taken within 1 month post-symptom presentation, within the 12 months before diagnosis. For women who had had multiple tests the authors only considered the first. This identified a population of symptomatic cervical cancers and associated cytology results. Because human papilloma virus (HPV) triage was not used during the study period, borderline results were divided into ‘borderline low risk’ for tests with an ‘early repeat’ action code (meaning that slides showed low-grade abnormalities and a repeat test was recommended) and ‘borderline high risk’ for tests with a ‘suspend’ action code (meaning that slides showed potentially high-grade disease and immediate colposcopy was recommended).

Box 1. **Gynaecological symptoms**

- Postcoital bleeding.
- Intermenstrual bleeding.
- Bleeding during pregnancy.
- Change in menstrual periods.
- Dyspareunia.
- Vaginal discharge.

A second estimate of sensitivity, and an indirect estimate of PPV, was obtained from the National Audit of Invasive Cervical Cancers.^18^^,^^19^ The authors used cytology results from women aged 20–24.5 years, diagnosed between 4 November 2010 (the date after which all women in England were first invited at age 25 years) and July 2014. Cytology in these women would not have been as a result of a screening invitation, and were likely taken in response to symptoms. For comparison, the authors also used audit data to obtain cytology results from all women with cervical cancer aged 20–29 years, diagnosed between April 2007 and July 2014. As the in-depth study falls within the same period (November 2010 to March 2012) as that of the women in the audit, the same women may appear in both datasets. However, it is possible that different cytology test results were used in the analysis. As audit data were provided anonymously, the authors were unable to identify which of the women appeared in both datasets.

Anonymous electronic primary care records obtained from the Clinical Practice Research Datalink (CPRD) database^8^ provided estimates for specificity (that is, the proportion of women without cervical cancer in the general population that would test cytology negative) and indirectly for PPV of cytology in women with gynaecological symptoms. The CPRD holds data from more than 600 primary care practices in the UK. The sample included consultation records for 45 484 women aged 20–29 years during the years 1990 to 2010. Consultation data were provided as medical codes and their associated dates. The authors identified women who had medical codes for gynaecological symptoms (Box 1), and codes related to cervical screening (for example, cytology results, diagnosis of cervical dysplasia, referral to colposcopy). Age at event, that is, at symptomatic presentation or cytology test, was taken to be the number of whole years between 1 July in the woman’s year of birth and the date of the medical code entry. The authors assumed consultation data were available for the entire period between the women’s first and last estimated age at event, even if no events were recorded in between. For example, if the first event was at age 21 years and the last event at age 28 years, the authors assumed they had data for ages 21–28 years. The authors estimated the proportion who presented with a gynaecological symptom when aged 20–29 years in 1-year age bands, and the proportion who had a cytology test taken within 31 days after symptomatic presentation.

Whole-population cytology from the national screening database provided data on all cervical cytology in England taken from April 2007 to March 2010 for women aged 20–29 years (that is, the general screened population). These data were collected in October and November 2010 for a different study.^20^ Only tests taken within the screening programme were included. This was used to estimate PPV directly.

### Analysis

The authors assessed the sensitivity of symptomatic cytology testing to cervical cancer for two thresholds: high grade, defined as borderline high risk, or moderate dyskaryosis, or worse; and very high grade, defined as severe dyskaryosis or worse (Table 1 provides a glossary of cytology results according to the British Society for Clinical Cytology (BSCC) and the nearest equivalent Bethesda system terminology).

**Table 1.** Glossary of cervical cytology results according to the British Society for Clinical Cytology (BSCC), and Bethesda terminology

**BSCC cytology result**	**Bethesda system cytology result**	**Diagnostic cytology thresholds**	
		**High grade^a^**	**Very high grade^b^**
Negative	Within normal limits		
Inadequate	Unsatisfactory		
Borderline change in squamous cells	ASC-US		
Borderline change, high-grade dyskaryosis not excluded	ASC-H	✓	
Borderline change in endocervical cells	AGC		
Mild dyskaryosis	LSIL		
Moderate dyskaryosis	HSIL	✓	
Severe dyskaryosis	HSIL	✓	✓
High-grade dyskaryosis ?invasive	Squamous cell carcinoma	✓	✓
?Glandular neoplasia	AIS	✓	✓

a High grade, defined as borderline high risk, or moderate dyskaryosis, or worse.

b Very high grade, defined as severe dyskaryosis or worse. AGC = atypical glandular cells. AIS = adenocarcinoma in situ. ASC-H = atypical cells of undetermined significance, cannot exclude a high grade squamous intraepithelial lesion. ASC-US = atypical cells of undetermined significance. HSIL = high-grade squamous intraepithelial lesion. LSIL = low grade squamous intraepithelial lesion.

PPVs of cytology were based on the first non-repeat (that is, not a follow-up test) cytology result within 12 months prior to diagnosis. The authors chose 12 months so as to allow for diagnosis following early (6-month) recall triggered by borderline or mild dyskaryotic cytology while trying to ensure that the cancer was already present at the time of cytology. Sensitivity analyses took the index test as the first within 9 and 18 months of diagnosis (data available from authors on request).

Direct estimates of PPVs were calculated by dividing the number of cancers diagnosed with a given index test result by the number of cytology tests with that result in the general population during the same period. Indirect estimates of PPV were calculated using the formula:

$$\mathrm{PPV}=\frac{\mathrm{sensitivity}\;\times \;\mathrm{prevalence}}{\mathrm{sensitivity}\;\times \;\mathrm{prevalence}+(1-\mathrm{specificity})\times (1-\mathrm{prevalence})}$$

The 95% confidence intervals for sensitivity and PPV were calculated assuming a binomial distribution. Confidence intervals for borderline low risk and borderline high risk took into account by simulation that the number of cytology tests in each of these categories in the general population was estimated. All analyses were carried out in Stata (version 13).

## RESULTS

### Sensitivity of cytology in young women with cervical cancer

In all, 22 of 107 women with primary care record data had a symptomatic cytology test (that is, first non-repeat test within 1 month of symptomatic presentation). Symptoms were postcoital bleeding (*n* = 17), intermenstrual bleeding (*n* = 6), vaginal discharge (*n* = 7), heavy or frequent periods (*n* = 4), and dyspareunia (*n* = 1). The sensitivity of high-grade cytology (borderline high risk, or moderate dyskaryosis, or worse) was 90.9% (95% CI = 70.8 to 98.8, and for very high grade (severe or worse) it was 81.8% (95% CI = 59.7 to 94.8) (Table 2). None of these 22 women had negative cytology.

**Table 2.** First non-repeat cytology test within 12 months prior to diagnosis for young women with cervical cancer for symptomatic cytology tests, and all cytology tests

	**Symptomatic cytology^a^**				**All cytology tests**	
	**Women aged 22–29 years diagnosed from Nov 2010 to Mar 2012 (in-depth study)**		**Women aged 20–24.5 years diagnosed from 4 Nov 2010 to Jul 2014 (national audit)**		**Women aged 20–29 years diagnosed from Apr 2007 to Jul 2014 (national audit)**	
	***n***	**%**	***n***	**%**	***n***	**%**
Negative	0	–	0	–	38	1.8
Inadequate	–	–	–	–	22	1.0
Borderline low risk	2	9.1	0	–	59	2.8
Mild dyskaryosis	0	–	1	3.8	68	3.2
Borderline high risk	1	4.5	2	7.7	107	5.0
Moderate dyskaryosis	1	4.5	1	3.8	187	8.8
Severe dyskaryosis	11	50.0	8	30.8	1185	55.8
?Glandular neoplasia	0	–	4	15.4	175	8.2
High-grade dyskaryosis ?invasive	7	31.8	10	38.5	282	13.3
**Total**	**22**	**100**	**26**	**100**	**2123**	**100**
High grade^b^	20	90.9	25	96.2	1936	91.2
Very high grade^c^	18	81.8	22	84.6	1642	77.3

a Women in the in-depth study had documented symptoms at the time of cytology. Those in the audit were presumed to be symptomatic at cytology, based on their age and the period examined.

b High grade, defined as borderline high risk, or moderate dyskaryosis or worse.

c Very high grade, defined as severe dyskaryosis or worse. Note: women from the in-depth study should also be included in the audit data because of the overlap in the period examined, but the cytology result used may not be the same.

From the national audit, 72 women aged 20–24.5 years were diagnosed between 4 November 2010 and July 2014. Of these, 36% (26 out of 72) had a cytology test before the first screening invitation (which was presumed to be in response to symptoms). The sensitivity of cytology for high grade was 96.2% (95% CI = 80 to 99), and 84.6% (95% CI = 65 to 96) for very high grade. Again, none of the women had negative cytology.

The sensitivity of cytology in symptomatic women was very similar to that in all women. Of 2463 women aged 20–29 years in the national audit diagnosed via screening or symptoms, 2123 had cytology within 12 months prior to diagnosis. The sensitivity of high-grade cytology was 91.2% (95% CI = 89.9 to 92.4), and 77.3% (95% CI = 75.5 to 79.1) for very high grade. There was no suggestion that the sensitivity of cytology to invasive cancer was less in symptomatic women than in screened women (Table 2).

### Specificity of cytology in symptomatic women for cervical cancer

In the CPRD dataset, the number of women available for study for each year of age ranged from 15 012 to 36 178. Within each single-year age band between 5.2% and 7.8% of women aged 20 to 29 presented to their GP with a gynaecological symptom (Appendix 1). The proportion of these with cytology was small (<14%). Cytology results were available for 1842 tests taken within a month of symptomatic presentation. Cytology was negative for 77.8% (86.2% of 1663 adequate samples) (Table 3). The distribution of results of the 1842 smears taken within 1 month of presenting with gynaecological symptoms was very similar to that of all other smears in the database: 2.0% (95% CI = 1.4 to 2.8) versus 1.8% (95% CI = 1.7 to 1.9) for moderate or worse, and 1% (95% CI = 0.6 to 1.5) versus 0.7% (95% CI = 0.67 to 0.81) for severe or worse, respectively. A further 10.5% had low-grade abnormalities (borderline or mild), and 9.7% of (mostly conventional) smears were classified as inadequate.

**Table 3.** Cytology results for symptomatic cytology versus all other cytology tests recorded in women aged 20–29 years (CPRD data 1990 to 2010)

**Cytology result**	**All other cytology,^a^*n* (%)**	**Symptomatic cytology,^b^*n* (%)**
Normal	47 175 (79.3)	1433 (77.8)^c^
Inadequate	4728 (7.9)	179 (9.7)
Borderline	3930 (6.6)	113 (6.1)
Mild dyskaryosis	2591 (4.4)	80 (4.3)
Moderate dyskaryosis	648 (1.1)	19 (1.0)
Severe dyskaryosis	431 (0.7)	18 (1.0)
?Glandular neoplasia	4 (0.01)	0 (0)
High-grade dyskaryosis ?invasive	7 (0.01)	0 (0)
**Total**	**59 514 (100)**	**1842 (100)**
Moderate dyskaryosis or worse	1090 (1.8)	37 (2.0)
Severe dyskaryosis or worse (very high grade)	442 (0.7)	18 (1.0)

a All other cytology results defined as not recorded within 1 month of presenting with a gynaecological symptom.

b Symptomatic cytology defined as test recorded within 1 month after presenting with a gynaecological symptom.

c If inadequate samples are excluded from the denominator, 86.2% (1433 out of 1663). CPRD = Clinical Practice Research Datalink.

### Positive predictive value of cytology testing for cervical cancer in young women

In the national audit data, 111 women aged 20–24 years, and 693 aged 25–29 years with cervical cancer had a non-repeat cytology test within 12 months of diagnosis, between April 2007 and March 2010 (Table 3). Over the same period, there were 258 425 non-repeat cytology tests in women aged 20–24 years in the general population (the majority of which would have been for screening, because invitations in this age group were still being phased out over this period) and 1 267 168 in women aged 25–29 years. As expected, for women aged 20–24 years, the majority of tests (82.0%) were negative, and 3.7% were high grade. The PPV of high-grade cytology for invasive cervical cancer was 1.01% (95% CI = 0.8 to 1.2). In women aged 25–29 years, the PPV was significantly greater at 1.74% (95% CI = 1.6 to 1.9). The PPV for very high grade was 2.0% (95% CI = 1.6 to 1.9) in women aged 20–24 years, and 3.15% (95% CI = 2.9 to 3.4) in women aged 25–29 years. It should be noted that the proportions of young women with borderline changes or mild dyskaryosis on cytology who have cervical cancer are very small at 0.04% to 0.05% (Table 4).

**Table 4.** Result of cytology by age group in women with cervical cancer (the first non-repeat test in the 12 months prior to diagnosis), and in the general population, and PPV of the result to cervical cancer

**Cytology test result**	**Tests and cancers between April 2007 and March 2010**				
	**Cancers**	**% cancers diagnosed with test result or worse**	**Cytology tests**	**% of all tests**	**PPV, %**
	**Age 20–24 years**				
Negative	0	100	211 930	82	0.00
Inadequate	0	100	7509	2.9	0.00
Borderline low risk	8	100	15 947	6.2	0.05
Mild dyskaryosis	6	93	13 440	5.2	0.04
Borderline high risk	8	87	2092	0.8	0.38
Moderate dyskaryosis	13	80	3722	1.4	0.35
Severe dyskaryosis	54	68	3622	1.4	1.49
?Glandular neoplasia	4	20	92	0	4.35
High-grade dyskaryosis ?invasive	18	16	71	0	25.35
**Total**	**111**		**258 425**		**0.04**
High grade^a^	97		9599		1.01
Very high grade^b^	76		3785		2.01
	**Age 25–29 years**				
Negative	17	100	1 108 469	87.5	0.00
Inadequate	7	98	32 223	2.5	0.02
Borderline low risk	22	97	51 910	4.1	0.04
Mild dyskaryosis	18	93	38 452	3.0	0.05
Borderline high risk	25	91	6809	0.5	0.37
Moderate dyskaryosis	68	87	12 301	1.0	0.55
Severe dyskaryosis	402	77	16 089	1.3	2.50
?Glandular neoplasia	60	19	595	0	10.08
High-grade dyskaryosis ?invasive	74	11	320	0	23.13
**Total**	**693**		**1 267 168**		**0.05**
High grade^a^	629		36 114		1.74
Very high grade^b^	536		17 004		3.15

a High grade, defined as borderline high risk or moderate dyskaryosis, or worse.

b Very high grade, defined as severe dyskaryosis or worse. PPV = positive predictive value.

Given the similar distribution of cytology results in symptomatic women to that in the general population (CPRD data, Table 3), one would expect the PPV among symptomatic women to be substantially greater. In the general population, the prevalence of (occult cytology-detectable) cervical cancer was between 0.04% and 0.05% (Table 4). Among women aged 20–29 years who consulted primary care (CPRD), between 5% and 8% had gynaecological symptoms (Appendix 1). According to interview data from the in-depth study, 89.0% (95% CI = 82.3 to 93.9) of women aged <30 years with cervical cancer reported having symptoms in the 2 years before diagnosis.^15^ Thus, the prevalence (% cancer in population × [proportion with symptoms/% symptoms in population]) of cervical cancer among symptomatic women is between 0.4% (0.04% × [0.82/8%]) and 0.9% (0.05% × [0.94/5%]). Using the usual formula,^21^ the PPV for cancer of high-grade cytology in symptomatic women would be between 10.0% and 30.0%.

## DISCUSSION

### Summary

These findings show that moderate or worse cytology (including borderline high risk) has high sensitivity (≥90.0%) for cervical cancer in young women with symptoms. Furthermore, none of the symptomatic women with cancer had negative cytology, implying that concerns over false reassurance from false-negative cytology tests are unfounded. Although the PPVs of symptomatic cytology tests in the screening population were 2.0% or less, these are likely to be substantially greater in symptomatic women with cervical cancer. The latest National Institute for Health and Care Excellence guidelines^12^ use a 3.0% PPV threshold for the suspected cancer referral pathway, although they state that lower thresholds should be used for young people. Therefore, it seems reasonable to suggest that severe or worse dyskaryosis in young women with gynaecological symptoms could prompt urgent referral, and non-urgent referral to colposcopy for moderate (or borderline, cannot exclude high grade) dyskaryosis. Cytology results of borderline or mild cytology (atypical cells of undetermined significance [ASCUS]/low-grade squamous intraepithelial lesion [LSIL]) would not prompt referral. However, in women with negative, borderline, or mild cytology thorough investigation of symptoms to explore other causes would still be warranted, as per usual care.

### Strengths and limitations

A key strength of the study is that the authors used cytology results for young women who were known to be symptomatic at the time. To the best of the authors’ knowledge these represent the first published data on the PPV for cancer of symptomatic cytology in young women.

Sensitivity estimates were very similar for women who were known to be symptomatic at the time of cytology testing (in-depth study) and those in the national audit. This is reassuring given that the authors only had data on a small number of women with symptoms and cannot be certain that women in the national audit data were symptomatic.

A drawback of the CPRD data is that the authors relied on clinical codes to identify symptomatic presentations. This is likely to have led to underestimates. Also, given that the analyses were based on retrospective review there is potential for confounding — for example, if women who received symptomatic cytology tests had a higher underlying risk of disease compared with symptomatic women who were not tested. However, as the sensitivity of cytology for cervical cancer in symptomatic women (in-depth study) was similar to that of women in the national audit (all cytology tests), any such confounding is likely to have been small.

Whole-population cytology from the national screening database was not recent (2007 to 2010), and may not accurately reflect the current situation. For example, some women aged 24 years at first cytology may have attended for routine screening and may not have been symptomatic.

### Comparison with existing literature

In a screening population of women aged 25– 64 years, the estimated PPV for cervical cancer of high-grade cytology was 2.0–5.0%, and 4.0–10.0% for very high grade.^22^ The authors’ estimate is much higher (10–30%), which is to be expected in a symptomatic population in whom the risk of disease is higher. With the imminent and expected switch to HPV primary screening in England, cytology triage could also become a useful tool in symptomatic women aged 25 to 64 years who test HPV negative, given that the screening interval could increase to >5 years.

Interview data from the in-depth study show that 60% of women aged <30 diagnosed with cervical cancer via symptomatic presentation experienced delays between first presentation and diagnosis.^15^ This suggests that a sizeable proportion of young women with cervical cancer could benefit from the use of cytology as a diagnostic aid. In addition, in the same study most of those with screen-detected cancer reported having symptoms on interview in the year before diagnosis.

### Implications for practice

Diagnostic cytology could help facilitate earlier diagnosis of cervical cancer by optimising triage of symptomatic young women in whom malignancy is unlikely but possible, alongside tests for genital infection. A substantial proportion of young women with cervical cancer stand to benefit. The majority (89%) retrospectively report having symptoms in the year before diagnosis,^15^ and, according to primary care records, 52% present with gynaecological symptoms.^14^

The authors believe that the viewpoint that cervical cytology should be restricted to screening is no longer tenable. These findings indicate that cytology could be an excellent diagnostic aid for detecting cervical cancer earlier in young women with symptoms. A large pilot implementation of such radically-revised management guidelines is warranted.

## Appendix 1. CPRD data (1990 to 2010) for proportion of women aged 20–29 years presenting to primary care with gynaecological symptoms, and the number of cytology tests taken within a month of symptomatic presentation with a result recorded

**Table:**

**Age at symptom, years**	**Number of women,^a^*n***	**Women who presented with a gynaecological symptom, *n*(%)**	**Smears taken within 1 month after presenting with a gynaecological symptom, *n*(%)**
20	36 178	1897 (5.2)	161 (8.5)
21	34 743	1898 (5.5)	160 (8.4)
22	32 998	1801 (5.5)	186 (10.3)
23	31 051	1796 (5.8)	197 (11.0)
24	29 006	1709 (5.9)	201 (11.8)
25	26 817	1666 (6.2)	214 (12.8)
26	24 064	1603 (6.7)	220 (13.7)
27	21 169	1553 (7.3)	177 (11.4)
28	18 150	1343 (7.4)	171 (12.7)
29	15 012	1172 (7.8)	155 (13.2)

a Number of women with consultation data available at each given age at symptom, assuming consultation data were available for the entire period between the age at the first and last recorded event. CPRD = Clinical Practice Research Datalink.
